# Adequacy and Acceptability of the Self-Collected Anal Pap Smear in People Living With Human Immunodeficiency Virus in the Infectious Diseases Clinic

**DOI:** 10.7759/cureus.58753

**Published:** 2024-04-22

**Authors:** Frederico Villa-Chan, Kellie Wark, Ryan Kubat, Jessica R Newman

**Affiliations:** 1 Internal Medicine, University of Kansas Medical Center, Kansas City, USA; 2 Infectious Diseases, University of Kansas Medical Center, Kansas City, USA

**Keywords:** infectious diseases clinic, hiv, hpv-related, pap smear, anal pap

## Abstract

Background

Anal Pap smears are imperative to screening for human papillomavirus (HPV)-associated anal squamous cell cancers, particularly in patients living with human immunodeficiency virus (HIV) given a higher incidence of disease. Self-collection of specimens may be favored by patients and more feasible to collect, increasing screening.

Methods

This was a single-center observational cohort study at a single academic medical center Infectious Diseases clinic from October to December 2021. We aimed to improve compliance of anal Pap collection documentation of “self-collected” versus “physician-collected” as well as verify if self-collected specimens (SCS) were adequate for interpretation equivalent to physician-collected specimens (PCS). Additionally, we aimed to evaluate patient and provider satisfaction with self-collected anal Paps.

Results

Sixty anal Pap smears were available for evaluation. The rate of documentation of the collection method (self-collected vs. physician-collected) was 88% during the intervention. A total of 75% of patients opted for self-collection, and 35/45 (78%) of these samples were adequate for interpretation. There was no difference in the adequacy of specimen (the ability of a cytopathologist to interpret the specimen) between the SCS and PCS.

Conclusion

Limited prior data suggest self-collected anal Pap specimens are adequate for interpretation only slightly less often than PCS. In our small cohort, there was no statistically significant difference between collection methods. Satisfaction with self-collection of specimens was high for both patients and providers. Additional validation in more diverse/larger clinical settings may be helpful to support this practice.

## Introduction

Anal Pap smears are important in screening for high-risk human papillomavirus (hrHPV)-associated anal squamous cell carcinoma (ASCC) as the anal cancer incidence has been found to be nearly 20 times higher for people living with human immunodeficiency virus (PLWH) compared to the general population [1,2]. Currently, there is no consensus regarding time intervals or protocols for screening for anal hrHPV-associated cancer in PLWH. The most recent IDSA (Infectious Diseases Society of America) guidelines (2020) recommend anal Pap smears in PLWH with a history of genital warts or in people with a history of receptive anal intercourse or abnormal cervical Pap smears [3]. The importance of screening has been demonstrated in recent studies, showing that treatment of high-grade anal dysplasia results in a lower rate of progression to anal cancer and that men diagnosed with anal cancer during screening have improved survival, likely due to diagnosis at an earlier stage [4,5]. While specific timing and procedures for specimen collection are not well-established, experts agree that if anal cytologic screening is performed, it should be followed with high-resolution anoscopy if anal dysplasia is noted [3,6].

For the assessment of sexually transmitted infections such as gonorrhea and chlamydia, self-collection of rectal specimens (SCS) for screening is well-accepted among patients and equivalent to physician collection as per Centers for Disease Prevention and Control (CDC) guidelines [7]. The best practice for collection of anal Pap smear collection has not been formally established. A crossover study from 2016 with women with HIV showed that the patients found self-performed tests to be feasible, and patients have found the practice highly acceptable [8]. In addition, the quality of samples has been found comparable to that of physician-collected specimens (PCS) [9]. A cross-sectional study with HIV-negative women performed in Puerto Rico in 2012 showed that even though most women preferred having a clinician collect anal samples (61%), the acceptability of both sampling methods (self-collected vs. clinician-collected) was high [10,11].

At our institution there was no standardization for documentation as to whether specimens were collected by patients or physicians, nor was there a standard education on best practices for specimen collection available for patients. It remained unclear what the adequacy of specimens collected by patients versus physicians was. It was also unclear if the practice of SCS would be well-accepted among patients and physicians in our Infectious Diseases clinic.

Our group designed a quality improvement project (QIP) with the goals of 1) improving compliance of anal Pap collection documentation of “self-collected” versus “physician-collected” to >70% over three months, 2) verifying no greater than 10% discrepancy in specimen adequacy (for interpretation) in SCS versus PCS over three months, and 3) improving patient and provider satisfaction with the anal Pap smear collection process. 

## Materials and methods

This was a single-center observational cohort study performed from October through December of 2021 in one academic medical center Infectious Diseases outpatient clinic. Educational material about the quality project background and aims was distributed by email to Infectious Diseases faculty and fellows (21), and nursing staff (three), before the start of the project. An educational flyer and reminder cards were posted in the Infectious Diseases clinic workroom near computer workstations during the project. Procedural handouts for patient education were produced and laminated (Figure 2 of Appendix). Patient educational handouts were housed in each clinic room and offered instructions for SCS for anal Paps. Information about the QIP was provided with this handout for patient review, and patients verbally consented by their physician at the time of specimen collection, which was performed with cytobrushes and Thin Prep vials with liquid medium [12]. Prior to the procedure, the patient’s physician reviewed the patient handout with them, and patients were given the opportunity to ask questions. All patients determined to benefit from anal Pap smear screening by their treating physician were included in the study if they consented, and all patients were offered self-collection if they preferred. Patient samples were excluded if they were collected outside of the study period or if they were not collected in the routine clinic rooms given the lack of availability of the patient handout and survey tool.

Nursing staff was asked at the time specimens were sent to the lab to enter a brief progress note for the patient with an electronic medical record (EMR) smart phrase containing the phrase #selfpap versus the phrase #drpap based on if the specimen was SCS or PCS, respectively. Infectious Diseases fellows and faculty were asked to add the EMR smart phrase to their visit progress note as well. These keywords were then used in the end-of-project data collection to tally SCS versus PCS in the data collection sheet.

At the beginning of the initial plan-do-study-act (PDSA) cycle, reminder emails were sent to the Infectious Diseases clinic faculty and staff about the initiation of the QIP. Early feedback showed that multiple providers missed documenting the smart phrases in their progress notes, highlighting the importance of the redundant entries by clinic nurses. In our second PDSA cycle, our team sent monthly reminders via email about the importance of documentation of the smart phrases. Tallies were made of the following for the pre- and post-intervention three months: SCS versus PCS (or not documented), specimen adequacy by a number of specimens adequate for interpretation (including a count of cytologic Bethesda Classification System interpretations as normal, atypical squamous cells of undetermined significance (ASCUS), atypical squamous cells, cannot exclude a high-grade squamous intraepithelial lesion (ASC-H), low-grade squamous intraepithelial lesion (LSIL), and high-grade squamous intraepithelial lesion (HSIL), and a number of specimens not adequate for interpretation (based on <2000 nucleated squamous cells and/or free from obscuring artifacts such as blood, excess bacteria, or fecal material) [13]. Satisfaction data was also tallied via a paper survey completed by patients at the time of their anal Pap smear collection and by providers at the conclusion of the intervention period of the QIP (Table 3 of Appendix).

## Results

At the conclusion of the intervention period, a total of 60 samples were available for evaluation. Encounters had the Pap smear collection modes appropriately documented 53/60 times (88%) compared to no documentation of the collection method pre-intervention. For the seven undocumented episodes, the treating physician was asked to verify the collection method for the purposes of comparison after the clinic encounter. Participating patients ranged in age from 20 to 72 years with a median of 44 years. All patients were cisgender male. A total of 60% of participants identified as white non-Hispanic (36/60), 30% were black non-Hispanic (18/60), 7% Hispanic (4/60), and 1.7% were Asian and bi-racial, respectively (1/60). Most participants (97%) reported English as their primary language and two were Spanish-speaking (3.3%). 

**Figure 1:**
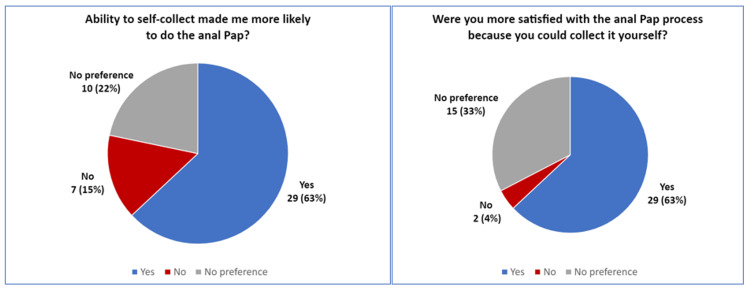
Patient Satisfaction Survey

**Figure 2:**
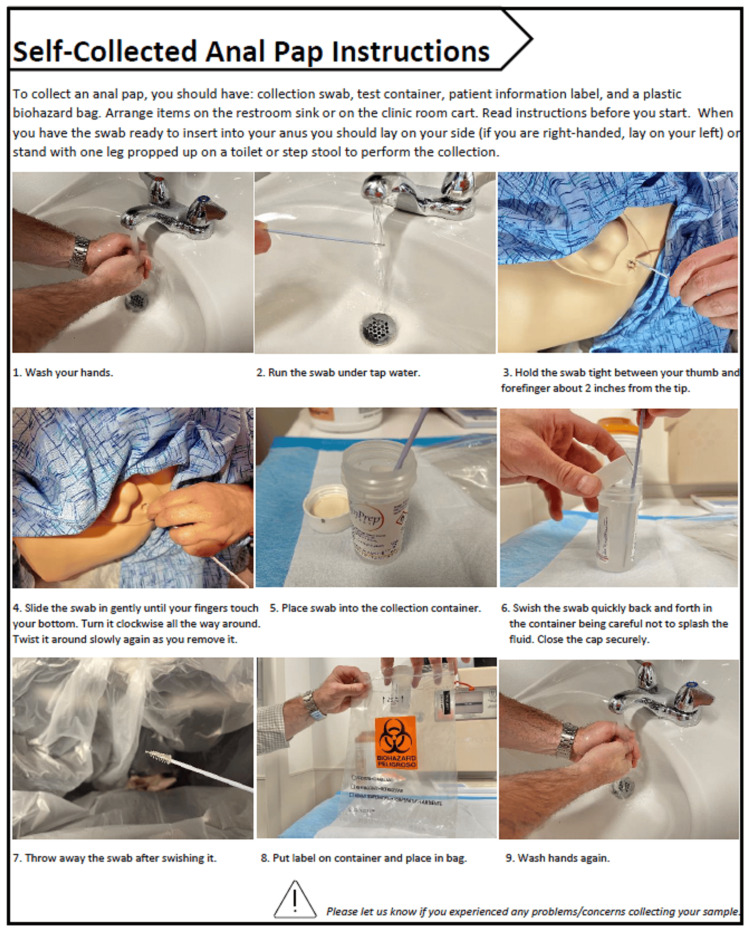
Self-Collected Anal Pap Patient Handout

A total of 75% of patients opted for self-collection and 35/45 (78%) of SCS were adequate for interpretation, compared to 10/15 (67%) of PCS. Fisher's exact test value was 0.49, indicating no significant difference between groups (at p<0.05). Of those adequate for interpretation, 19 were normal, 14 were ASCUS, one was ASC-H, seven were LSIL, and three were HSIL. The study was not powered to draw conclusions on differences in these interpretations between groups. In total, 77% of patients completed the satisfaction survey. A total of 63% were more satisfied with the anal Pap process because they could self-collect, whereas 33% had no preference and 4% were less satisfied (Figure 1). Only 13% reported a preference for physician-performed procedures, while 63% felt that a self-collection option made them more likely to agree to complete the Pap that visit (Figure 1). For those performing SCS, the reasons they preferred to self-collect were cited as increased privacy (26/46) and less discomfort (11/46).

For physicians, the response rate for the survey was 95% (20/21 physicians). In total, 90% of physicians or 19/21 felt more satisfied with the anal Pap procedure because the patients could collect it (Table 1).

**Table 1:** Physician Satisfaction ^1^One respondent answered both “yes” and “no preference.”

Satisfaction	Yes (%)	No (%)	Neutral (%)
Greater satisfaction with anal Pap procedures because the patient could self-collect them^ 1^	19/21 (90%)	1/21 (5%)	2/21 (10%)
Comfort with offering patients the option to self-collect anal Pap swabs	18/21 (86%)	1/21 (5%)	2/21 (10%)

The majority (76%) of respondents favored patient collection with an additional 19% having no preference. Also, 86% of providers felt comfortable offering the patients the option to self-collect. Of concerns cited, 12/21, or 60% felt some concern prior to data collection about an inadequate sample with patient collection (Table 2).

**Table 2:** Physician Preference

Preference	Self (physician, %)	Patient (%)	Neutral (%)
Who would you prefer to collect the patient’s anal Pap smear swabs	1/21 (5%)	16/21 (76%)	4/21 (19%)
Concerns	Yes (%)		
Inadequate sample	12/21 (57%)		
Patient acceptance of self-collection	4/21 (19%)		
Potential costs if self-collection is inadequate sample	3/21 (14%)		

## Discussion

Self-collection of anal Pap smears appears safe with no significant adverse events reported in the reviewed studies or in our project. Limited reports suggest SCS are adequate for interpretation only slightly less often than PCS with a similar sensitivity for detection of abnormal cytology [14,15]. Satisfaction with the SCS was high.

Overall, our results suggested that there was no significant difference in the adequacy of SCS versus PCS. Like prior studies, our patients were satisfied with the self-collection and the majority felt this option made them more likely to complete the test at their visit. While the project was not powered to demonstrate an increase in acceptance of anal Paps, increased patient satisfaction could lead to improvement over time with a simple and inexpensive workflow adjustment. The option of self-collection also expands options for timing of SCS such as in between visits, which may be especially useful given the increased uptake in telehealth care delivery. Also, if physicians are not required for screening completion, additional collections could take place in non-physician office settings such as private or commercial laboratories, and this increased flexibility could have the benefit of increased screening. We also found high satisfaction with the self-collection from the physician's perspective.

There are several limitations to this study. First, it occurred at a single Infectious Diseases clinic at one University-based academic hospital. Thus, the applicability to other clinic settings or larger practices may be limited. Studies with larger populations, in multiple institutions and with varying educational handouts/instruction methods, may provide better data to corroborate recommendations regarding best practices for screening for ASCC. Additionally, some providers preferred self- or physician collection due to current practices prior to the project, which may have introduced bias into the orientation to the self-collection procedures to the patient. While all physicians were aware of general procedures for specimen collection and these were provided via educational handouts at the beginning of the project, no formal training or verification of procedural capabilities was verified in the context of the QIP. Additionally, the Hawthorne effect may have influenced results, as participants were aware of the QIP, and that the validity of the samples depended on appropriate sample collection. There was also a relatively small sample size of total specimens (n=60). 

## Conclusions

Screening for hrHPV-associated ASCC, particularly in PLWH, is imperative. We found that self-collection of anal Pap can successfully be completed in the clinic. The self-collected anal Pap is as adequate as physician-collected and acceptable to patients and physicians. Due to the high acceptance of this practice overall, the clinic now routinely offers SCS, and procedural patient information handouts are still utilized in patient rooms. Handouts have been distributed to other primary physicians in our center providing care for PLWH, and steps have been taken to extend the offering of self-collection to the main outpatient laboratory.

## Appendices

**Table 3:** Patient Satisfaction Survey

Were you MORE satisfied with your anal Pap because you could collect it yourself?	Yes	No	No preference
Who would you prefer collect your anal Pap swab?	Me	Doctor	No preference
Did being offered to collect your own swab make you MORE likely to do the Pap?	Yes	No	No preference
If you prefer collecting your own swab, please circle reasons why (can circle more than one)	More private	Less uncomfortable	More confident in test result

## References

[1] Colón-López V, Shiels MS, Machin M (2018). Anal cancer risk among people with HIV infection in the United States. J Clin Oncol.

[2] Shiels MS, Cole SR, Kirk GD, Poole C (2009). A meta-analysis of the incidence of non-AIDS cancers in HIV-infected individuals. J Acquir Immune Defic Syndr.

[3] Thompson MA, Horberg MA, Agwu AL (2021). Primary care guidance for persons with human immunodeficiency virus: 2020 update by the HIV Medicine Association of the Infectious Diseases Society of America. Clin Infect Dis.

[4] Palefsky JM, Lee JY, Jay N (2022). Treatment of anal high-grade squamous intraepithelial lesions to prevent anal cancer. N Engl J Med.

[5] Van der Zee RP, Wit FWNM, Richel O, van der Valk M, Reiss P, de Vries HJC, Prins JM (2023). ATHENA national observation HIV cohort. Effect of the introduction of screening for cancer precursor lesions on anal cancer incidence over time in people living with HIV: a nationwide cohort study. Lancet HIV.

[6] Panel on Guidelines for the Prevention and Treatment of Opportunistic Infections in Adults and Adolescents with HIV. (2023). Guidelines for the Prevention and Treatment of Opportunistic Infections in Adults and Adolescents With HIV. National Institutes of Health, Centers for Disease Control and Prevention, HIV Medicine Association, and Infectious Diseases Society of America. National Institutes of Health, Centers for Disease Control and Prevention, HIV Medicine Association, and Infectious Diseases Society of America.

[7] Workowski KA, Bachmann LH, Chan PA (2021). Sexually Transmitted Infections Treatment Guidelines, 2021. MMWR Recomm Rep.

[8] Heid-Picard B, Cochand-Priollet B, Rozenberg F, Giang-Phang D, Viard JP, La Torre V, Ghosn J (2021). Ambulatory anal self-sampling in MSM living with HIV, an acceptable and reliable screening method. PLoS One.

[9] McNeil CJ, Kong CS, Anglemyer A, Levy V, Maldonado Y (2016). Results of the women’s self-performed anal Pap trial in human immunodeficiency virus-infected women. Sex Transm Dis.

[10] Ortiz AP, Alejandro N, Pérez CM (2012). Acceptability of cervical and anal HPV self-sampling in a sample of Hispanic women in Puerto Rico. P R Health Sci J.

[11] Ortiz AP, Romaguera J, Pérez CM (2013). Human papillomavirus infection in women in Puerto Rico: agreement between physician-collected and self-collected anogenital specimens. J Low Genit Tract Dis.

[12] Darragh TM, Jay N, Tupkelewicz BA, Hogeboom CJ, Holly EA, Palefsky JM (1997). Comparison of conventional cytologic smears and ThinPrep preparations from the anal canal. Acta Cytol.

[13] Pangarkar MA (2022). The Bethesda System for reporting cervical cytology. Cytojournal.

[14] Cranston RD, Darragh TM, Holly EA (2004). Self-collected versus clinician-collected anal cytology specimens to diagnose anal intraepithelial neoplasia in HIV-positive men. J Acquir Immune Defic Syndr.

[15] Lampinen TM, Latulippe L, van Niekerk D, Schilder AJ, Miller ML, Anema A, Hogg RS (2006). Illustrated instructions for self-collection of anorectal swab specimens and their adequacy for cytological examination. Sex Transm Dis.

